# Removal of metallic tracheobronchial stents in lung transplantation with flexible bronchoscopy

**DOI:** 10.1186/1749-8090-5-72

**Published:** 2010-09-12

**Authors:** Oren Fruchter, Yael Raviv, Benjamin D Fox, Mordechai R Kramer

**Affiliations:** 1 The Pulmonary Institute, Rabin Medical Center, Beilinson Campus, Petah Tiqwa, Israel

## Abstract

**Background:**

Airway complications are among the most challenging problems after lung transplantation, and Self-Expandable Metallic Stents (SEMS) are used to treat airway complications such as stenosis or malacia at the bronchial anastomosis sites. Several transplantation centers are reluctant to use SEMS since their removal is sometimes needed and usually requires the use of rigid bronchoscopy under general anesthesia. The objective of the current report is to describe our experience in SEMS retrieval by flexible bronchoscopy under conscious sedation.

**Methods:**

A retrospective review was done of patients requiring tracheobronchial stent placement after lung transplantation in which the SEMS had to be removed. The retrieval procedure was done by flexible bronchoscopy on a day-care ambulatory basis.

**Results:**

Between January 2004 and January 2010, out of 305 lung transplantation patients, 24 (7.8%) underwent SEMS placement. Indications included bronchial stenosis in 20 and bronchomalacia in 4. In six patients (25%) the SEMS had to be removed due to excessive granulation tissue formation and stent obstruction. The average time from SEMS placement to retrieval was 30 months (range 16-48 months). The stent was completely removed in five patients and partially removed in one patient; no major complications were encountered, and all patients were discharged within 3 hours of the procedure. In all procedures, new SEMS was successfully re-inserted thereafter.

**Conclusions:**

The retrieval of SEMS in patients that underwent lung transplantation can be effectively and safely done under conscious sedation using flexible bronchoscopy on a day-care basis, this observation should encourage increasing usage of SEMS in highly selected patients.

## Introduction

Airway complications are a significant source of morbidity and mortality among lung transplantation patients. The reported incidence of airway complications following lung transplantation varies widely from 1.6% to 33% according to different reports [1-6]. Self-Expandable Metallic Tracheobronchial Stents (SEMS) are inserted with increasing frequency in patients that underwent lung transplantation and developed airway complications such as anastomotic dehiscence, excessive refractory granulation tissue formation and stenosis at the bronchial anastomosis [5-8]. The main advantage of SEMS is the ease of their placement, a procedure that usually can be performed using flexible bronchoscope under conscious sedation. Another significant advantage is the low incidence of stent migration. The major complication of SEMS is the obstruction of the stent by excessive granulation tissue [6-8]. The granulation tissue can usually be treated by various endoscopic modalities such as mechanical detriment and laser photoresection, but occasionally stent removal is mandatory. Fracture of the stent wires may rarely occur and also requires removal of the stent. The endoscopic retrieval of SEMS is technically demanding, and unfortunately, is reported to be associated with significant complications (retained stent, mucosal tear, bleeding, reobstruction, and pneumothorax) [9,10]. In contrast to silicone stents that can be relatively easily removed by pulling, the removal of SEMS usually requires the use of rigid bronchoscopy and optical forceps since the stent is embedded within the bronchial wall. A major disadvantage of using rigid bronchoscopy is the need for general anesthesia, that may discourage lung transplantation centers from using SEMS for treating airway complications in their high anesthetic risk patients [11-13].

We wish to describe our experience in removing SEMS deployed in patients that underwent lung transplantation under conscious sedation using flexible bronchoscopy based techniques without the need for rigid bronchoscopy.

## Subjects and methods

We retrospectively analyzed the clinical course of all lung transplant recipient from January 2004 until January 2009 at the Rabin Medical Center, a tertiary-care medical center with an average volume of 50 lung transplantation per year. Airway complications, indications for treatment with SEMS, and indications for removal were analyzed. Institutional review board approval was obtained, but specific informed consent was not required for this retrospective study. Informed consent for each bronchoscopy was obtained prior to the procedure. The routine protocol for Immunosuppression in our institute includes tacrolimus, mycophenolate mofetil and glucocorticoids.

The SEMS used were SMART nitinol stent, (Cordis, Miami, FL, USA), Wallstent (Boston Scientific Corp; Natick, MA, USA), or LUMINEX (BARD; Germany). Before retrieval of the SEMS each patient underwent a standard pre-operative assessment, including physical examination, routine laboratory tests, spirometry, and chest radiography and computed tomography of the chest. Before the decision to extract the SEMS was made previous techniques to manage restenosis and granulation tissue formation were attempted in all patients that included mechanical debridement and Nd: YAG Laser photoresection (SHARPLAN-3000, SHRPLAN Lasers)

**Figure 1:**
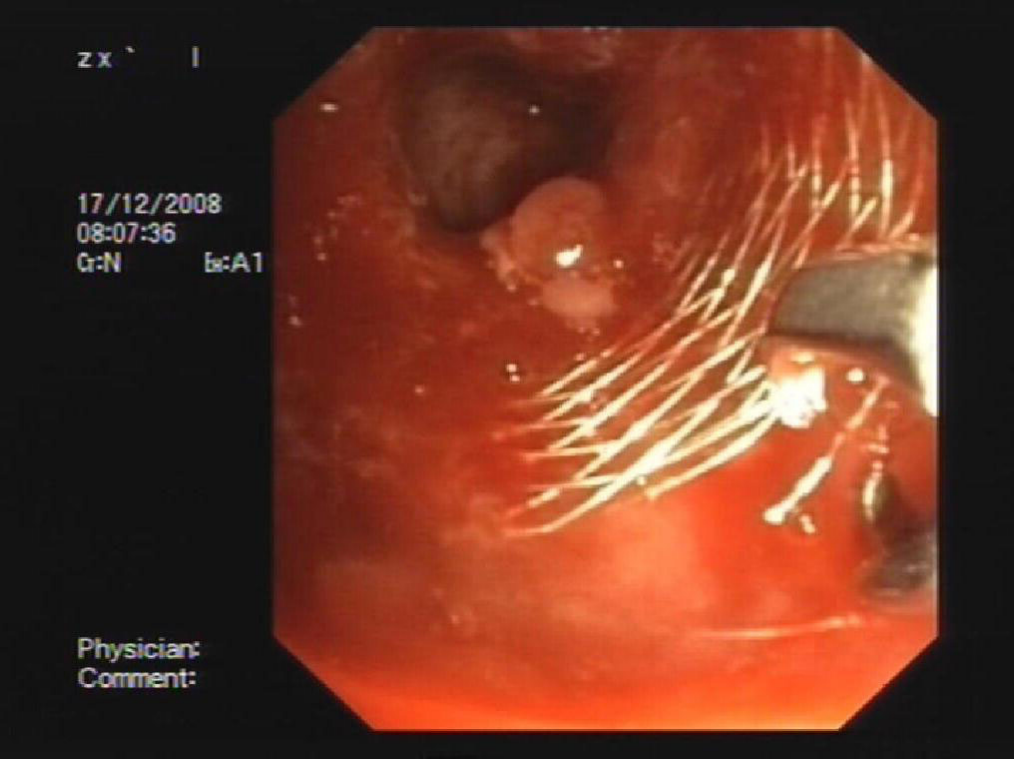
**A representative figure of stent removal in patient # 4 in whom biopsy forceps are applied during retrieval of 12 × 40 mm Self-Expandable Metallic Stent from the right main stem bronchus**. The patient had underwent lung transplantation for idiopathic pulmonary fibrosis.

All bronchoscopic retrieval procedures were done on an ambulatory day-care setting. To provide analgesia and sedation, midazolam (1-10 mg) and alfentanyl (0.5-1.5 mg) were administrated. If deemed necessary, supplemental doses of propofol (20 mg) were administered at an interval of 2-5 minutes.The procedures were carried out with supplemental O2 through nasal cannula. In all procedures, bronchoscopy was performed using a large working channel bronchoscope (Olympus Excera; BF-1TQ180 II video endoscope, Olympus, Tokyo, Japan). Standard biopsy forceps were used to carefully dissect the stent from the airway wall. Next, the proximal portion of the stent was grasped and steady traction was applied until the entire stent, or part of the stent was removed (See figure 1). This approach was repeated if necessary until all stent fragments were removed. Following removal of the stent the entire airway lumen was assesed for bleeding and other complications such as mucosal tear and retained secretions. All patients were observed for two hours and discharged following chest-x-ray.

## Results

Over 5-year period, 305 lung transplantations were performed at our institute. Out of these patients, 24 (7.8%) had underwent uncovered SEMS placement. The types of SEMS used were Wallstent (Boston Scientific), SMART (Cordis Johnson & Johnson), and LUMINEX (BARD). Indications included bronchial stenosis in 20, and bronchomalacia in the remaining 4 patients. The overall granulation tissue formation rate mandating treatment to prevent re-stenosis was 65%. We noted no differences between SEMS types with respect to the complications associated with them and the resulting indication for removal. In Six patients (25%) the SEMS had to be removed due to excessive granulation tissue formation. Patient's demographic and clinical characteristics are presented on Table 1. The average age was 60.2 years, and the average time from SEMS placement to retrieval was 30 months (range 16-48 months). The indication for removal of the stent was excessive granulation formation and refractory stenosis of the bronchial lumen. All retrieval procedures were done by flexible bronchoscopy under moderate sedation via the trans-oral approach using a bite-block. The average procedure time was 25 minutes (range 12-65 minutes). In 2 cases (cases # 1 and # 3) endotracheal tube was inserted electively to facilitate the oral removal of the stent by the flexible bronchoscope forceps.

**Table 1:** Demographic and Clinical characteristics of the study patients.

*#*	*Gender/age*	*Indication* *for lung Tx*	*FEV_1 _before SEMS* *Placement *	*FEV_1_* *after* *SEMS* *placement *	*SEMS type location and size (mm)*	*Interval from lung Tx to SEMS* *insertion* *(months)*	*Interval from insertion to retrieval* *(months)*	*Outcome*
1	M/64	IPF	45%	58%	Wallstent; RMSB 12/40	6 m	16 m	Complete removal/replacement with new SEMS

2	M/62	IPF	43%	66%	Wallstent; LMSB 12/40	1 m	18 m	Partial removal/replacement with new SEMS

3	F/46	LAM	53%	71%	Wallstent; LMSB 12/40	5 m	48 m	Complete removal/replacement with new SEMS

4	M/64	IPF	66%	71%	Wallstent; RMSB 12/40	5 m	33 m	Complete removal/replacement with new SEMS

5	M/70	COPD	56%	78%	Luminex; LMSB 35/9	2 m	39 m	Complete removal/replacement with new SEMS

6	M/57	COPD	68%	72%	Smart; RMSB 14/40	13 m	24 m	Complete removal/replacement with new SEMS

**Abbreviations**: FEV1: Forced expiratory volume in first second percent of predicted; RMSB: right main stem bronchus;LMSB: left main stem bronchus; LAM: lymphangioleiomyomatosis; IPF: Idiopathic pulmonary fibrosis; COPD: Chronic obstructive pulmonary disease; Lung Tx: lung transplantation

All patients had successful endoscopic extraction of the stents. In a single patient-(case # 2), small retained pieces of the stent could not be removed although they did not disturb airway patency. In all procedures, a new SEMS was successfully re-inserted following removal of the previous stent. Upon a follow up period (median 14 months), apart from granulation tissue formation that mandated repeated Nd:YAG Laser photoresection, there was no need to extract the newly positioned SEMS. Complications of stent removal were uncommon and included, mild mucosal bleeding. No patient developed pneumothorax or pneumomediastinum. No patient needed mechanical ventilation or hospitalization following the procedure, and all patients were discharged within 3 hours of the procedure.

## Discussion

The incidence of airway complications mandating SEMS placement in our series (7.6%) is similar to that previously reported by several transplantation centers [1-5]. In lung transplantation patients, SEMS are used to treat various airway complications ranging from anastomotic and non-anstomotic bronchial stenosis, and dehiscence [5-8]. Granulation tissue formation is common following SEMS placement although the incidence is lower compared to that reported in patients in whom SEMS were placed for other benign conditions. This complication often requires repetitive interventions to treat granulomas and sometimes require stent removal. The incidence of stent related complications mandating removal in the current series (25%) is lower than that reported the retrieval of metallic stents is sometimes complicated and several authors suggest that it should be accomplished only by rigid bronchoscopy [9-13]. A major disadvantage in the use of rigid bronchoscopy is the need for general anesthesia. In the current series although uncuffed endo-tracheal tube was inserted in 2 cases to facilitate orotracheal removal of the SEMS, no general anesthesia was used and the patients were spontaneously breathing under conscious sedition throughout the entire procedure. We noted no differences between the SEMS types and the ease of their removal. Similarly, we noted no significant differences in retrieval success between SEMS that had been placed several months earlier and those that had been recently placed and needed to be removed.

Several previous reports addressed the issue of endoscopic removal of SEMS. Unfortunately most studies have addressed the issue of SEMS extraction in malignant patients or in other benign conditions and not in lung transplantation patients. The largest series to date is by Lunn et al [12] that describes their experience in retrieval of 30 SEMS using rigid bronchoscopy. However, none of these patients had undergone lung transplantation. Noppen and colleagues [13] describe their experience in removal of 10 SEMS in patients with benign airway disorders. In that series removal of the stent was also performed with rigid bronchoscopey in a fully equipped operating theater under total IV anesthesia. Similar to the previous series by Lunn et al [12] none of the subjects was a lung transplant patient. The experience of removal of SEMS placed in patients that underwent lung transplantation is very limited. Sonnets at al. [14] described a case report of a patient that underwent removal of a SEMS in a patient that underwent double lung transplantation and developed bronchial stenosis. Mughal et al [15] described 2 lung transplant patients that underwent flexible bronchoscopic removal of SEMS under conscious sedation placed for bronchial dehiscence. The major difference is that in that report the median time from stent placement to removal was short (37 days) as the SEMS were used as a temporary measure to treat bronchial dehiscence.

To the best of our knowledge, our study is the largest to date to describe the endoscopic removal of SEMS placed in patients that underwent lung transplantaion. It is unique in several aspects. First, we chose not to use rigid bronchoscopy in this particular group of patientsto obviate the need for general anesthesia. In all cases the procedure was accomplished under moderate sedation on an ambulatory day-care basis which has proved to be a cost-effective intervention obviating the need for a costly operating theater and hospitalization [16]. General anesthesia may be problematic in lung transplantation patients with compromised airways and performance of the procedure under spontaneous ventilation was proved to be safe. Additional difference of the current report is that the median period between stent placement and removal in our series is significantly longer than that reported previously. Lunn et al. [12] has shown that the rate of complications during stent removal is directly related to the time the stent has been in place. The longer the stent is situated in the airway it tends to adhere to the bronchial wall making its removal theoretically more difficult. In the current series we noted no such trend for increased complications rate proportional to the time the stent has been in place.

Whereas a previous report have suggested that the use of SEMS in lung transplantation patients should be restricted to a minimum due to excessive granulation tissue formation [9], we [17] have noted that lung transplantation patients tend to develop less granulation tissue than other patients. We speculate that the use of immunosuppressive agents in these patients may reduce the formation of granulation tissue formation compared to non-transplanted patients with stents.

Our experience as has been described in the current series is encouraging since it highlights the reversibility of SEMS placement. We believe that his observation should encourage increasing usage of SEMS in these patients in whom airway complications are common and are a major cause for post transplantation morbidity. However, given the high complications rate in their removal as reported by previous authors [9-14], their use however must be very cautious and as the last resource.

The practical implication of the current report is that SEMS can be used more liberally in highly selected lung transplantation patients since their removal, if found to be necessary, could be safely and effectively accomplished using flexible bronchoscopy under conscious sedation on a day-care basis.

## Competing interests

The authors declare that they have no competing interests.

## Authors' contributions

OF: Participated in the design of the study and performed the Clinical work. YR: Participated in the design of the study and performed the Clinical work, BF: the Clinical work. MK: Participated in the design of the study and performed the Clinical work. All authors read and approved the final manuscript.
